# Can Early Post-Operative Scoring of Non-Traumatic Amputees Decrease Rates of Revision Surgery?

**DOI:** 10.3390/medicina60040565

**Published:** 2024-03-30

**Authors:** Vesta Brauckmann, Ole Moritz Block, Luis A. Pardo, Wolfgang Lehmann, Frank Braatz, Gunther Felmerer, Sebastian Mönnighoff, Jennifer Ernst

**Affiliations:** 1 Department of Trauma Surgery, Hannover Medical School, Carl-Neuberg-Straße 1, 30625 Hannover, Germany; brauckmann.vesta@mh-hannover.de; 2 Department of Trauma Surgery, Orthopedic Surgery and Plastic Surgery, University Medical Center, 37075 Göttingen, Germanyluis.pardo@med.uni-goettingen.de (L.A.P.J.); wolfgang.lehmann@med.uni-goettingen.de (W.L.); frank.braatz@med.uni-goettingen.de (F.B.); gunther.felmerer@med.uni-goettingen.de (G.F.); 3 Orthobionics Study Programme, Private University of Applied Sciences, 37073 Göttingen, Germany; sebastian.moenninghoff@t-online.de

**Keywords:** lower-limb amputation, non-traumatic amputation, revision surgery, scoring system

## Abstract

*Background and Objectives:* Medical registries evolved from a basic epidemiological data set to further applications allowing deriving decision making. Revision rates after non-traumatic amputation are high and dramatically impact the following rehabilitation of the amputee. Risk scores for revision surgery after non-traumatic lower limb amputation are still missing. The main objective was to create an amputation registry allowing us to determine risk factors for revision surgery after non-traumatic lower-limb amputation and to develop a score for an early detection and decision-making tool for the therapeutic course of patients at risk for non-traumatic lower limb amputation and/or revision surgery. *Materials and Methods*: Retrospective data analysis was of patients with major amputations lower limbs in a four-year interval at a University Hospital of maximum care. Medical records of 164 patients analysed demographics, comorbidities, and amputation-related factors. Descriptive statistics analysed demographics, prevalence of amputation level and comorbidities of non-traumatic lower limb amputees with and without revision surgery. Correlation analysis identified parameters determining revision surgery. *Results:* In 4 years, 199 major amputations were performed; 88% were amputated for non-traumatic reasons. A total of 27% of the non-traumatic cohort needed revision surgery. Peripheral vascular disease (PVD) (72%), atherosclerosis (69%), diabetes (42%), arterial hypertension (38%), overweight (BMI > 25), initial gangrene (47%), sepsis (19%), age > 68.2 years and nicotine abuse (17%) were set as relevant within this study and given a non-traumatic amputation score. Correlation analysis revealed delayed wound healing (confidence interval: 64.1% (47.18%; 78.8%)), a hospital length of stay before amputation of longer than 32 days (confidence interval: 32.3 (23.2; 41.3)), and a BKA amputation level (confidence interval: 74.4% (58%; 87%)) as risk factors for revision surgery after non-traumatic amputation. A combined score including all parameters was drafted to identify non-traumatic amputees at risk for revision surgery. *Conclusions*: Our results describe novel scoring systems for risk assessment for non-traumatic amputations and for revision surgery at non-traumatic amputations. It may be used after further prospective evaluation as an early-warning system for amputated limbs at risk of revision.

## 1. Introduction

Despite limb-saving innovations in medicine, the latest available numbers in Germany indicate an increase of 3.5% in lower limb amputations, with a total number of 57,637. The number of minor amputations have been increasing along with a decrease in major lower limb amputation [1,2]. This may be due to improved prevention, education and medical technologies in interventional angiography and vascular surgery [3,4,5,6,7]. The aetiology of amputations can be manifold. However, complications of diabetes mellitus remain the most frequent underlying reason and possibly the reason why this subgroup of amputation was shown to be associated with complications rates up to 50%, and often followed by further revision surgeries [8,9]. Revision rates for lower amputations range from 25–40% [9], with one third of revision cases resulting in a higher amputation level [1,10,11]

A crucial point for the success and course of rehabilitation is primary wound healing and early prosthetic fitting enabling mobility. This requires the determination of the right amputation level considering biomechanical principles and patient-based context factors [10,12,13]. Assessing the need for revision in early stages is necessary to achieve the best possible postoperative outcome. It has been shown that revisions lead to a delay in the patient’s postoperative mobility and rehabilitation [14]. 

In previous studies, residual limb pain and/or phantom limb pain, late infection, symptomatic bone spurs, revision skin grafts and improvement of the residual limb for prosthetic fitting were described the main indications for revision surgery [15,16,17,18,19]. Risk factors significantly associated with early complications after major lower extremity amputation include initial emergency surgery, transmetatarsal amputations as compared to below-knee amputations (BKA), sepsis, septic shock or SIRS, involvement of a novice at surgery, final renal disease, Body Mass Index (BMI) ≥ 30, and current nicotine use [20]. Furthermore, a significant association between a prolonged hospital stay until amputation and a higher revision rate has been shown [21].

Several studies have already described the epidemiological data on major amputations in Germany [1,2] and at our maximum care provider location [22]. However, data depicting the longitudinal course of non-traumatic amputations and conclusions to allow for early-stage identification criteria for amputees at risk of revision surgery are lacking.

The purpose of this study was to evaluate a retrospective data set from a maximum care provider to determine risk factors for revision in non-traumatic lower-extremity amputation, and to use this information to develop a revision score for early detection of affected patients and provide a decision-making tool on the further therapeutic course. 

## 2. Materials and Methods

### 2.1. Study Design

Ethical approval for this study was granted by the local ethics committee. The retrospective study part collected data from the intra-hospital database (SAP) using following amputation-related international diagnosis code (ICD) and the German official operation and procedure coding system (OPS).

See Table 1: OPS Procedure Codes. See Supplementary Data S1: All ICD-Codes.

After identifying the amputation statistics from 2013–2016 for lower limb amputation, amputation at the tarsometatarsal level or proximal level in one or both lower limbs were included for further analysis.

### 2.2. Retrospective Data Analysis

The selected study group included 164 patients, the medical records of which were requested. Data collected from the medical records included:

- Sociodemographic data (age at amputation, sex, height, weight);
- Amputation-related data (date of admission, amputation, and discharge; total duration of hospital stay; level and side of amputation, caring postoperative ward);
- ICD-coded comorbidities;
- Revision-related data (revision date, number of revisions, time (days) between revisions, reason for revision, performed surgical procedure);
- Other (documented wound healing disorders, infection, ulceration, bleeding, nicotine use, alcohol consumption).

All data was rendered pseudonymous and tabulated in an amputation registry on a spreadsheet database (Microsoft Excel 2020, Microsoft Corporation, Redmond, Washington, DC, USA).

### 2.3. Analysis of ICD-Coded Comorbidities

In the analysed timeframe all comorbidities documented in the intra-hospital database were first extracted. Then, non-traumatic amputee comorbidities occurring in >30% of the study subgroup were included in a second table for further analysis. (See Supplementary Data S2: Included ICD Codes)

### 2.4. Processing of the ICD-Coded Comorbidities and Hypothesized Risk Factors

The cumulative comorbidities grouped under the headings of lifestyle, neuropathic foot, vascular disease, and infection were listed. Priorly described and risk factors hypothesized by us, such as the length of hospital stay before amputation, time from admission to amputation, postoperative ward (surgical vs. non-surgical), and BMI, were added to our design of an amputation score [8,12,23,24]. It is worth noting that smoking was not an identified risk factor after the above described analysis but was still added to the score as it is commonly described as a risk factor for amputation [24]. Then, maxima and minima, as the confidence intervals, were calculated. 

From this table, the revised non-traumatic amputees were extracted and the type, aetiology of revisions and differentiation between early and late complications was added to the first draft of the revision score. Next, a graphical evaluation of the longitudinal course per amputee was performed, followed by a comparison f the initial amputation level until revision led to a stable, defined as no documentation for revision surgery or re-amputation within the analysed timeframe, amputation level..Confidence intervals were calculated for the time (days) from the first revision and the time to the final amputation level.

Correlation analyses on revisions were performed by comparing the calculated confidence intervals for comorbidities of non-traumatic amputees, the post-operative ward, days until amputation, and amputation level distal to the knee. Correlation analysis comparing the calculated confidence intervals of amputation related factors was performed to investigate whether general risk factors also apply to a higher risk of revision surgery. The graphical evaluation of the longitudinal course per amputee revealed the risk factor days to amputation > 30. Subsequently, the parameters were weighted by a factor of 0–3 based on their prevalence in the analysed cohort. Thereby, identified risk factors for revision surgery entered the score. For the design of the non-traumatic amputation revision score, the non-traumatic amputation score was completed with the identified relevant comorbidities and risk factors for revision surgery.

The designed revision score was applied in both subgroups of the study, lower limb amputees with and without revision surgery, to identify a cut-off value. The results are presented graphically using box plots.

## 3. Results

### 3.1. Demographics and Amputation Level Non Traumatic Amputees

**Figure 1:**
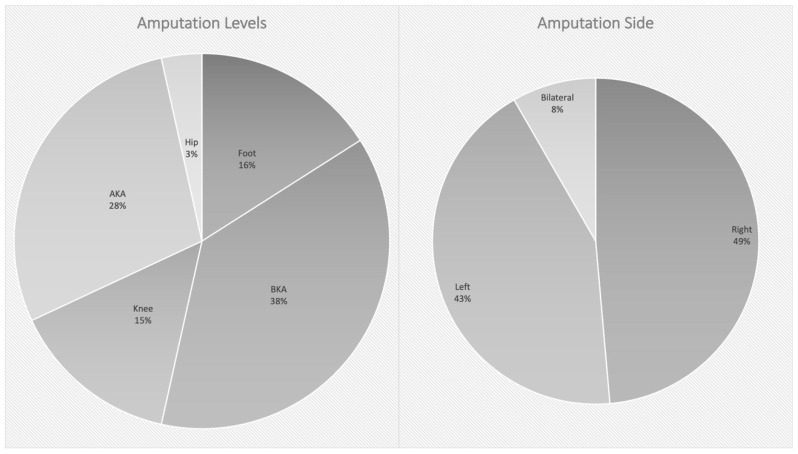
Level and side in non-traumatic amputees.

In total, 164 patients between 2013 and 2016 suffered 199 major amputations and 121 minor amputations of the lower limb. A total of 72% (N = 118) were male and 28% (N = 46) were female, with a mean age of 68 years (range between 19 and 97 years old). A total of 144 patients (88%) were amputated for non-traumatic reasons and 20 patients (12%) due to trauma. In the non-traumatic subgroup, 23 patients underwent amputation of the foot, 54 below the knee (BKA), 21 of the knee, 41 above the knee (AKA) and 5 of the hip. In total, there were 70 patients with an amputation on the right side, 62 on the left, and 12 bilateral amputations. See Figure 1.

### 3.2. Demographics and Amputation Level of Revised Non Traumatic Amputees

A total of 39 (27.1%) non-traumatic amputees were revised, with a total number of 63 revisions. Of the 39 revised patients, 66.7% (N = 26) were male and 33.3% (N = 19) were female. Mean age was 68.2 years, with the youngest being 39 and the oldest 94. A total of 14 (36%) patients had an initial amputation at the distal level of the foot, 15 (38%) BKA and 7 (18%) at the knee and 3 patients (8%) had an AKA amputation. In total, the revisions were performed among 19 right-sided, 13 left-sided and 7 bilaterally amputated patients.

Of the 39 revised non-traumatic amputees, 22 patients received one revision, 17 patients received two revisions, 4 patients three revisions, 2 patients four revisions, and 1 patient five revisions.

The following shifts in amputation level were observed after the first revision led to a stable residual limb during the observed time: 4 (10%) foot, 10 (26%) BKA, 9 (23%) knee, 14 (36%) AKA, and 2 (5%) hip.

The amputation level shifted proximally until the final revision except for 29% of the foot and 67% of the BKA non-traumatic amputees. After the revision interventions, there were two more post-amputations at knee level and number of AKA levels increased almost fivefold compared to the initial amputation level. Two amputees received a stable residual limb after being revised to hip disarticulations. This level was not present at first amputation. This means 35.9% (confidence interval (21.2%; 52.82%)) of the original BKA level (74.4%, confidence interval (57.87%; 86.96%)) could preserve the knee in this non-traumatic amputation subgroup.

The 63 revisions were due to the following: debridement (*n* = 23, 37%), re-amputation (*n*= 37, 59%), neuroma (1, 0.02%), pain (*n* = 1), wound healing disorder (*n*= 42), infection (*n* = 11), necrosis (*n* = 5), hematoma (*n* = 2). A total of 77% of the revisions were performed during the initial hospital stay (early complications), and 23% were discharged and revised in a second hospital stay. Please see Table 2.

### 3.3. Prevalence of ICD-Coded Comorbidities in Non-Traumatic Amputees

A total of 183 comorbidities were recorded. The diagnoses that occurred in the less-than-five patients excluded reduced the number of documented comorbidities to 32. (See Supplementary Datas S2 and S3). 

### 3.4. Prevalence of ICD-Coded Comorbidities of Not Revised Non-Traumatic Amputees

Lifestyle: 24 (17%) non-traumatic amputees smoked, 60 (42%) were diagnosed diabetes, and 16 had diabetes (11%) together with hyperlipidaemia. Furthermore, in the amputation score, a BMI above 26 was set as relevant (confidence interval: (26.1; 28.5)). Neuropathic foot: 16 (11%) of the non-traumatic amputees suffered polyneuropathy. Vascular diseases: 103 non-traumatic amputees (72%) suffered from pAVD, 99 (69%) from atherosclerosis and 55 (38%) had hypertension. Thrombosis, embolism, or myocardial infarction was documented in the history of 27 (19%). Infection: Sepsis or SIRS was documented for 28 non-traumatic amputees (19%), wound healing disorders for 37 (26%), fasciitis for 4 (3%), infection in 18 (13%), and osteomyelitis occurred in 13 (9%). A total of 46 (32%) of non-traumatic amputees had ulceration, and 68 (47%) suffered from gangrene.

### 3.5. Prevalence of ICD-Coded Comorbidities of Revised Non-Traumatic Amputees

To investigate which comorbidity determines revision, the coded comorbidities in the subpopulation of patients undergoing revision were analysed and the following results were obtained. Lifestyle: the BMI of nontraumatic patients averaged 28.6 kg/m^2^ (confidence interval: (26.3; 30.9)); thus, there was a 95% probability for a patient to be revised if suffering from pre-adiposity. Vascular disease: 29 patients (74%) suffered from PAD and 28 (72%) suffered from atherosclerosis. Arterial hypertension has been reported in 14 (36%). *Infection*: 27 patients (69%) suffered from wound healing disorders.

See Table 3. Risk factors of patients in correlation to revisions.

### 3.6. Duration of Hospital Stay of Not-Revised Non-Traumatic Amputees

The shortest stay within the subgroup of all non-traumatic amputees was 1 day and the longest 156 days. The mean length of stay was 43 days (CI: (37.9; 47.6)). The minimal interval between admission to amputation was 0 days, i.e., amputation on the admission day. The maximum interval was 141 days. On average, 19 days passed until amputation (CI: (15.9; 22.8)). 

### 3.7. Duration of Hospital Stay of Revised Non-Traumatic Amputees

Within the subgroup of revised non-traumatic amputees, the minimum time between amputation and first revision was one day; the maximum 220 days. On average, 34 days passed (CI: (20; 47.3)). The minimum duration from amputation to last revision was 4 days; the maximum duration was 281 days. On average, 44 days passed until the last revision. (CI: (27.8; 60)).

### 3.8. Results of the Correlation Analyses of Revision Surgery vs. Risk Factors

A second analysis examined whether the comorbidities that are identified risk factors for amputation are also risk factors for revision surgery. Correlation analysis of the following risk factors covering lifestyle, neuropathic foot, vascular disease, and infection showed the following correlations.

Lifestyle risk factors were not significantly related to revisions. A significant association with revision was found for wound healing disorders in the infection subgroup (Table 2: Risk factors of patients in correlations to revisions). Revisions were significantly more frequent on surgical wards (CI: 0.6 (0.55; 0.82)) than on external non-surgical wards (CI: 0.4 (0.22; 0.54)). Considering the inpatient stay from admission to amputation, a positive correlation between the number of days to amputation and revision was shown. Revised patients had an average hospital stay of 32.3 days (CI: (23.2; 41.3)) and non-revised of 14.5 days (CI: (11.7; 17.4)). 

### 3.9. Non-Traumatic Amputation Score

Following the results of the study, the first design of an predictive non-traumatic amputation grouped under the headings of lifestyle, neuropathic foot, vascular disease and infection included the following parameters: pAD (72%), arterial hypertension (38%), diabetes (42%), overweight defined as BMI > 25 (confidence interval: (26.1; 28.5)), the presence of gangrene (47%), sepsis (19%), and age older than or equal to 68.2. Smoking has been found to be significant in the literature and was also included in the score [25].

These risk factors were evaluated based on the absolute percentage in the subgroup or defined stages (e.g., PAD) weighted by a factor of 0–3, resulting in the following score.

### 3.10. Non-Traumatic Revision Score

Correlation analysis revealed that wound healing disorders occurred in 64.1% (confidence interval (47.18%; 78.8%)) of revised and 11.43% (confidence interval (6.05%; 19.11%) of non-revised non-traumatic amputees. The mean hospital stay was 32.3 days before initial amputation in the revised group (confidence interval (23.2;41.3)) in contrast to 14.5 (confidence interval (11.7; 17.4)) in the non-revised one. Of the revised non-traumatic amputees, 74.4% (confidence interval (57.87%; 86.96 %)) initially had an amputation distal to the knee (BKA), and the non-traumatic amputees without revision, and 59% (confidence interval (49.02%; 68.55%)) initially had an amputation below the knee (BKA). Finally, the non-traumatic amputation score was extended to the parameters wound healing disorder, distal amputation and time between admission and amputation, as these factors correlated positively with a revision surgery and were therefore considered risk factors. Furthermore, some of them may add a predictive character to the score.

See Table 4: Non-Traumatic Amputation score and Revision risk score.

### 3.11. Identification of the Cut Off-Values for the Designed Scoring Systems

The non-traumatic amputation score was applied to the study population and differentiated between the revision and the non-revision subgroup. For the amputation score, the interquartile range of patients without revision was higher than of patients with revision. The mean value of patients with revision was 4.9 points (CI: (4.2; 5.6)) and of patients without revision 5.4 points (CI: (4.9; 5.9)) Thus, the non-traumatic amputation score is not sufficiently discriminating for the identification of non-traumatic amputees at risk of revision surgery.

The non-traumatic amputation score was thus shown to not be sufficient in the identification of revision patients. Significant results were shown for the non-traumatic revision score (three parameters wound healing disorder, distal amputation and time between admission and amputation) and the combined amputation–revision score. (See Table 4).

**Figure 2:**
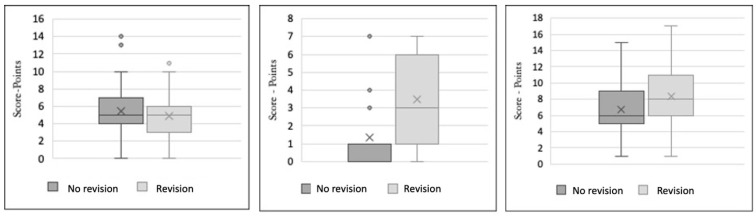
(**Left**): Boxplot of amputation score depending on revisions. Two subgroups were formed after calculating the values of the score corresponding to revised and non-revised patients: (a) no revision; (b) with revision. (**Center**): Boxplot of amputations, revision risk score in relation to revisions. Two subgroups were formed after calculating the values of the score corresponding to revised and non-revised patients: (a) no revision; (b) with revision. (**Right**): Boxplot of combined amputation-revision score depending on revisions. Two subgroups were formed after calculating the values of the score corresponding to revised and non-revised patients: (a) no revision; (b) with revision. The revision risk score can be used as a rapid form for already-amputated patients and the extended score as an extensive predictive score before amputations.

The interquartile range of revised patients was shown to be higher than of patients without revision. For the non-traumatic revision score, the mean value of patients with revision was 3.5 points (CI: (2.82; 4.12)) and of patients without revision 1.3 points (CI: (1.05; 1.62)). For the combined amputation–revision score, the mean value of revised patients was 8.4 points (CI: (7.34; 9.45) and of patients without revision 6.7 points (CI: (6.16; 7.31). This means that the isolated added parameters showed non-traumatic amputees with and without revisions (See Figure 2). 

In combination, both scores (see Table 4) showed a significant difference in the scores of the revised non-traumatic amputees. A cut-off value of a lower limb or residual limb at risk of revision surgery could be set at 7 points based on the confidence intervals. This cut-off value was composed of a comparison of the calculated mean values of the revised non-traumatic amputees versus the non-revised traumatic amputees.

## 4. Discussion

The purpose of this study was to generate a dataset for the improved and specific evaluation of the revision risk after amputation, with particular attention on the treatment after non-traumatic amputation of the lower limbs. To the best of our knowledge, such a score does not exist for non-traumatic amputation. Meta-analyses evaluating scores predicting wound healing for diabetic foot ulcers and/or amputation report variable rates of both among patients with incident diabetic foot ulceration. They reflect a poor quality of studies and a pressing need for standardized individual participant data to provide powerful data that can be combined across sites to maximize precision, examined for heterogeneity, and adjusted for potential confounding factors and develop prognostic utility for the prediction of healing. A total of 9476 publications were reviewed. Among them, the measurement of skin perfusion pressures, toe pressures and TcPO_2_ appear seem to be more useful in predicting ulcer healing and amputation than ankle pressures or the ABI [26].

Established scores to classify wound severity (e.g., Wound, Ischemia, and foot Infection (WIfI) classification) seem to correlate with the risk for amputation [27] but are not validated to predict the risk for revision surgery or re-amputation after wound healing occurred after amputation. A recent research article concluded that the early identification and treatment of patients at high risk for wound chronicity and complications, followed by early referral to and treatment at a specialized wound clinic, resulted in faster healing and reduced health system costs [28]. Re-amputation or revision surgery after amputation is a complication which sets back functional rehabilitation, often leads to protracted hospitalization, and is associated with significant morbidity and mortality. Many attempts failed over the years to predict wound healing after amputation, but, despite this, re-amputation rates remain high [29]. Smoking and elevated white blood cells were described to be associated with a higher risk for re-amputation in non-traumatic amputees with wound healing [30,31].

In our study collective, there were on average 80 amputations per 41 patients per year, with 20–40% of revisions. The gender and age distribution in our cohort was comparable with described epidemiology in the previous literature [2,7]. In our score, we included PAD, diabetes, hypertension, BMI, nicotine, wound complications, and age.

We found that 38% of our collective were treated for hypertension, which leads us to wonder if the endovascular damage of hypertension may play a bigger role than previously assumed. An example for scores described in previous research are the Charlson Comorbidity Index [25], or more specific to amputation the AMPREDICT Mobility score for probability of mobility and independence after dysvascular amputation [14]. Common comorbidities of the scores are myocardial infarction, diabetes, and higher age. The AMPREDICT Score set sex, BMI, high blood pressure, lung disease, dialysis, stroke, and nicotine abuse as further relevant factors determining mobility after amputation and it needs to be evaluated if those correlate with increased revision rates, too.

Lifestyle factors play an important role in postoperative treatment; in particular, a BMI >26 kg/m^2^ increases mortality and rehabilitation after amputation [7,14]. Contrary to the literature, we found no correlation between obesity or smoking in our subgroup. Maybe this is due to a lower percentage of documented smokers in our subgroup [7,20,32]. Interestingly, other associated risk factors also differed in percentage in our study from previous literature. Polyneuropathy has been described in 43% of amputated patients, while in our study collective only 11,1% suffered from polyneuropathy. At the same time peripheral vascular disease (PVD) or thromboembolic events were found to be comparable with 71.5% for PVD to 43–76% described in the literature and 19% for thromboembolic events to 7–28% described in the literature [14,33,34]. Not much focus in research has been put on hypertension and amputation.

Since would healing disorders (infection, sepsis, osteomyelitis, ulcerations, gangrene) are not only described as leading cause of amputation, but for revision [7,34], it was of no surprise that it was found in about one fifth (26%) of our non-traumatic amputation cohort and in 69% of the revisions.

Sepsis was found in about 20% in our cohort compared to 32–37% in previous research [7]. Osteomyelitis was found in 9% of non-traumatic amputations and ulceration in 32%. In preceding studies, the numbers were 5.6% for osteomyelitis and 33% for ulceration. Surprisingly, infection in our study population was lower with 13%, as compared to the literature (28%) [33,34]. Our results do not appear to corroborate pain and soft-tissue pathologies as main reasons for revision. The reason might be the short follow-up times and thus less reported late complications [15,16]. Another reason might be that the study population might not be prosthetised. It is critical to note that while we found a distal amputation level to be a risk factor for revision, a more proximal amputation level has been previously associated with overall increased mortality [35].

The average time between admission and amputation was 19 days, with an average total stay of 43 days. The in-hospital mortality, risk of in-hospital death and the average recovery times have been shown to increase with longer until amputation [36]. This finding might be related to a correlation of time and an increased risk for relevant infections [37]. These findings underline the need for a quick decision-making for non-traumatic amputations and their revisions to decrease hospital stay and mortality.

The limitations of our study are partially incomplete datasets due to digitalization of files after 2015 and possible incomplete documentations of comorbidities and treatment of late complications in different clinics. Another limitation is that we focused on comorbidities, not including perioperative medication. A limitation of the non-traumatic revision score is the lacking predictive character, especially of the second part. To gain significance, wound healing disorder was defined as a non-healed residual limb 21 days after surgery. The aim should be to revise it earlier to reduce secondary complications due to delayed immobilization [32] and to indicate the right amputation level at first amputation [33,34]. Furthermore, we should consider a closer look at parameters impairing wound, especially when amputation is performed below the knee. This could be nutrition, local perfusion, and skin quality at the level of the residual limb. In future research, the score should be expanded to include larger cohorts in a prospective analysis for a reliable validation of the score and cut-off values.

## 5. Conclusions

The data obtained identified wound healing disorders, >30 days from admission to amputation and an amputation below the knee as risk factors for revision for non-traumatic amputees.

Based on the results, a score was developed, with significant results for patients at risk of revision. The scores yield significant results based on the patient population studied. Future prospective and multicentric research to validate the combined amputation–revision score is needed and should include a standardized collection of data. The findings suggest that this approach may be used as an early warning system for amputated limbs at risk of revision and to reduce secondary complications, and ultimately the numbers of revision numbers, through distribution of available resources and thorough aftercare.

## Figures and Tables

**Table 1:** English translation of OPS Procedure Codes; OPS = Operation and Procedure Coding System for different amputation levels of the lower limb from the level of hemipelvectomy to the tarsometatarsal level.

OPS-Code	Procedure
5-864	Amputation and disarticulation of the lower limb
5-864.0	Hemipelvectomy
5-864.1	Incomplete hemipelvectomy
5-864.2	Hip disarticulation
5-864.3	Above the knee amputation, not specified
5-864.4	Amputation of the proximal femur
5-864.5	Amputation of the shaft or distal femur
5-864.6	Amputation on knee-level
5-864.7	Knee disarticulation
5-864.8	Below the knee amputation, not specified
5-864.9	Below the knee amputation, proximal
5-864.a	Below the knee amputation, mid
5-865	Amputation and Disarticulation of the foot
5-865.0	Syme-Amputation
5-865.1	Amputation of the foot, not specified
5-865.2	Spitzy-Amputation
5-865.3	Pirogoff-Amputation
5-865.4	Chopart-Amputation
5-865.5	Lisfranc-Amputation

**Table 2:** Demographics, amputation level, reason for revision and type of revision in non-traumatic amputees.

	Total N = 39 (100%)
Male	26 (66.7%)
Female	19 (33.3%)
Age at amputation (mean, range in years)	68.2 (39–94)
Revision amputation level	
Foot	14 (36%)
BKA	15 (38%)
Knee	7 (18%)
AKA	3 (8%)
Reason for Revision	Total of Revisions N = 63
Pain	1 (0.02%)
Would Healing Disorder	42 (0.67%)
Infection	11 (17.5%)
Hematoma	2 (0.04%)
Necrosis	5 (0.08%)
Type of Revision	
Debridement	23 (37%)
Re-Amputation	37 (59%)
Neuroma Resection	1 (0.02%)

**Table 3:** Risk factors of patients in correlation to revisions (CI = Confidence Interval, PAD = peripheral artery disease, SIRS = Systemic Inflammatory Response Syndrome).

Risik Factor	Revision Yes (N = 39)	Revision No (N = 105)	Total (N = 144)
Lifestyle			
BMI	28.55 % (26.3; 30.8)	26.79 (25.4; 28.2)	27.28 (26.1; 28.5)
Nicotine consumption	15.38% (5.86%; 30.53%)	17.14% (10.49%; 25.73%)	16.67% (10.98%; 23.78%)
Diabetes	46.15% (30.09%; 62.82%)	40% (30.56%; 50.02%)	41.67% (33.52%; 50.17%)
Hyperlipidaemia	12.82% (4.3%; 27.43%)	10.48% (5.35%; 17.97%)	11.11% (6.49%; 17.42%)
Neuropathic Foot			
Polyneuropathy	10.26% (2.87%; 24.22%)	11.43% (6.05%; 19.11%)	11.11% (6.49%; 17.42%)
Vascular disease			
PAD	76.92% (60.67%; 88.87%)	69.52% (59.78%; 78.13%)	71.53% (63.42%; 78.73%)
Atherosclerosis	71.79% (55.13%; 85%)	67.62% (57.79%; 76.43%)	68.75% (60.5%; 76.21%)
Hypertonus	35.9% (21.2%; 52.82%)	39.05% (29.67%; 49.06%)	38.19% (30.23%; 46.65%)
Documented thrombosis/embolism	28.21% (15%; 44.87%)	23.81% (16.04%; 33.11%)	25% (18.16%; 32.89%)
Heart attack	17.95% (7.54%; 33.53%)	19.05% (12.04%; 27.87%)	18.75% (12.73%; 26.1%)
Infection			
Sepsis/SIRS	17.95% (7.54%; 33.53%)	20% (12.83%; 28.93%)	19.44% (13.33%; 26.86%)
Wound healing disorder	64.1% (47.18%; 78.8%)	11.43% (6.05%; 19.11%)	25.69% (18.78%; 33.64%)
Infection	20.51% (9.3%; 36.46%)	9.52% (4.66%; 16.82%)	12.5% (7.58%; 19.03%)
Ulceration	33.33% (19.09%; 50.22%)	31.43% (22.72%; 41.22%)	31.94% (24.43%; 40.22%)
Gangrene	28.21% (15%; 44.87%)	54.29% (44.28%; 64.04%)	47.22% (38.85%; 55.71%)
Osteomyelitis	0% (0%; 9.03%)	12.38% (6.76%; 20.24%)	9.03% (4.89%; 14.94%)
Fasciitis	2.56% (0.06%; 13.48%)	2.86% (0.59%; 8.12%)	2.78% (0.76%; 6.96%)

**Table 4:** Combined Non-Traumatic Amputation/Revision Score: 1. Non-Traumatic Amputation score and 2. Revision risk score (PAD = peripheral artery disease).

Amputation Score				
				Value:
1.	PAD	I–III:	+1	
		IV:	+2	
		Closure:	+3	
2.	Hypertension	Yes	+1	
		No	+0	
3.	Diabetes	Yes	+2	
		No	+0	
4.	BMI	25.0–29.9	+1	
		30.0–34.9	+2	
		35.0–39.9	+3	
		>40	+4	
5.	Smokers	Yes	+1	
		No	+0	
6.	Gangrene/necrosis of the foot		+1	
	Sepsis		+2	
7.	Age	<55	+0	
		56–75	+1	
		>75	+2	
	Maximum score value:		15	
Revision risk score				
8.	Postoperative wound healing disorders	Yes	+3	
	(3 weeks post-op not healed)	Neo	+0	
10.	Time between admission and amputation	>30 Days	+3	
		<30 Days	+0	
11.	Amputation distal to the knee	Yes	+1	
		No	+0	
	Maximum score value:		7	
Combined non-traumatic amputation-revision score				
	Maximum score value		22	

## Data Availability

The data that support the findings of this study are available from the corresponding author, upon reasonable request.

## Supplementary Materials

The following supporting information can be downloaded at: https://www.mdpi.com/article/10.3390/medicina60040565/s1, Supplementary Data S1: All ICD-Codes. Supplementary Data S2: Included ICD-Codes. Supplementary Data S3. Amputation levels from initial non traumatic amputation to fifth revision.
