# Supplementary material for: Can Early Post-Operative Scoring of Non-Traumatic Amputees Decrease Rates of Revision Surgery?
Source: Medicina (Kaunas). 2024 Mar 30;60(4):565. doi: 10.3390/medicina60040565 (PMC11052005; doi:10.3390/medicina60040565)
Supplement: Supplementary file 1 [file medicina-60-00565-s001.zip › medicina-2830817-supplementary.pdf]

ICD-Codes	Diagnose
A41.0	Sepsis durch Staphylococcus aureus
A41.9	Sepsis, nicht näher bezeichnet
A49.0	Staphylokokkeninfektion nicht näher bezeichneter Lokalisation
B95.6	Staphylococcus aureus als Ursache von Krankheiten, die in anderen Kapiteln klassifiziert sind
B96.2	Escherichia coli [E. coli] und andere Enterobakteriazeen als Ursache von Krankheiten, die in anderen Kapiteln klassifiziert sind
C04.9	Bösartige Neubildung des Mundbodens, nicht näher bezeichnet
C44.7	Sonstige bösartige Neubildungen der Haut der unteren Extremität, einschließlich Hüfte
D62	Akute Blutungsanämie
D68.9	Koagulopathie, nicht näher bezeichnet
E10.40	Diabetes mellitus, Typ 1 : Mit neurologischen Komplikationen : Nicht als entgleist bezeichnet
E10.50	Diabetes mellitus, Typ 1 : Mit peripheren vaskulären Komplikationen : Nicht als entgleist bezeichnet
E11.41	Diabetes mellitus, Typ 2 : Mit neurologischen Komplikationen : Als entgleist bezeichnet
E11.50	Diabetes mellitus, Typ 2 : Mit peripheren vaskulären Komplikationen : Nicht als entgleist bezeichnet
E11.51	Diabetes mellitus, Typ 2 : Mit peripheren vaskulären Komplikationen : Als entgleist bezeichnet
E11.60	Diabetes mellitus, Typ 2 : Mit sonstigen näher bezeichneten Komplikationen : Nicht als entgleist bezeichnet
E11.73	Diabetes mellitus, Typ 2 : Mit multiplen Komplikationen : Mit sonstigen multiplen Komplikationen, als entgleist bezeichnet
E11.75	Diabetes mellitus, Typ 2 : Mit multiplen Komplikationen : Mit diabetischem Fußsyndrom, als entgleist bezeichnet
E11.90	Diabetes mellitus, Typ 2 : Ohne Komplikationen : Nicht als entgleist bezeichnet
E11.91	Diabetes mellitus, Typ 2 : Ohne Komplikationen : Als entgleist bezeichnet
E14.74	Nicht näher bezeichneter Diabetes mellitus : Mit multiplen Komplikationen : Mit diabetischem Fußsyndrom, nicht als entgleist bezeichnet
E14.90	Nicht näher bezeichneter Diabetes mellitus : Ohne Komplikationen : Nicht als entgleist bezeichnet
E66.02	Adipositas durch übermäßige Kalorienzufuhr : Adipositas Grad III (WHO) bei Patienten von 18 Jahren und älter
E78.5	Hyperlipidämie, nicht näher bezeichnet
F10.2	Psychische und Verhaltensstörungen durch Alkohol : Abhängigkeitssyndrom
G20.90	Primäres Parkinson-Syndrom, nicht näher bezeichnet : Ohne Wirkungsfluktuation
G63.2	Diabetische Polyneuropathie (icd E10-E14 Diabetes mellitusE10-E14, vierte Stelle .4+)
G82.29	Paraparese und Paraplegie, nicht näher bezeichnet : Nicht näher bezeichnet
G93.1	Anoxische Hirnschädigung, anderenorts nicht klassifiziert
I10.00	Benigne essentielle Hypertonie : Ohne Angabe einer hypertensiven Krise
I25.13	Atherosklerotische Herz-Kreislauf-Krankheit, Drei-Gefäß-Erkrankung
I25.19	Atherosklerotische Herz-Kreislauf-Krankheit, Nicht näher bezeichnet
I38	Endokarditis, Herzklappe nicht näher bezeichnet
I50.9	Herzinsuffizienz, nicht näher bezeichnet
I70.20	Atherosklerose, Becken-Bein-Typ, ohne Beschwerden - Stadium I nach Fontaine
I70.21	Atherosklerose, Becken-Bein-Typ, mit belastungsinduziertem Ischämieschmerz, Gehstrecke 200 m und mehr - Stadium IIa nach Fontaine
I70.22	Atherosklerose, Becken-Bein-Typ, mit belastungsinduziertem Ischämieschmerz, Gehstrecke weniger als 200 m - Stadium IIb nach Fontaine

I70.23	Atherosklerose, Becken-Bein-Typ, mit Ruheschmerz - Stadium III nach Fontaine
I70.24	Atherosklerose, Becken-Bein-Typ, mit Ulzeration - Stadium IV nach Fontaine mit Ulzeration Gewebedefekt begrenzt auf Haut [Kutis] und Unterhaut [Subkutis]
I70.25	Atherosklerose, Becken-Bein-Typ, mit Gangrän - Stadium IV nach Fontaine mit GangränTrockene Gangrän, Stadium IVa nach Fontaine - Feuchte Gangrän, Stadium IVb nach Fontaine
I70.29	Atherosklerose, Sonstige und nicht näher bezeichnete Atherosklerose der Extremitätenarterien - [pAVK] ohne Angabe eines Stadiums (nach Fontaine)
I70.9	Atherosklerose, Generalisierte und nicht näher bezeichnete Atherosklerose
I73.1	Sonstige periphere Gefäßkrankheiten - Thrombangiitis obliterans [Endangiitis von-Winiwarter-Buerger]
I74.2	Embolie und Thrombose der Arterien der oberen Extremitäten
I74.3	Embolie und Thrombose der Arterien der unteren Extremitäten
I77.8	Sonstige näher bezeichnete Krankheiten der Arterien und Arteriolen - Arrosion Arterie - Ulkus Arterie
I79.2	Periphere Angiopathie bei anderenorts klassifizierten Krankheiten - Periphere diabetische Angiopathie
I82.9	Embolie und Thrombose nicht näher bezeichneter Vene
I83.1	Varizen der unteren Extremitäten mit Entzündung
I87.21	Venöse Insuffizienz (chronisch) (peripher) mit Ulzeration
I89.0	Lymphödem, anderenorts nicht klassifiziert
J44.99	Chronische obstruktive Lungenkrankheit, nicht näher bezeichnet : FEV1 nicht näher bezeichnet
J90	Pleuraerguss, anderenorts nicht klassifiziert
L02.4	Hautabszess, Furunkel und Karbunkel an Extremitäten
L03.02	Phlegmone an Zehen
L03.11	Phlegmone an der unteren Extremität
L58.9	Radiodermatitis, nicht näher bezeichnet
L89.34	Dekubitus 4. Grades : Kreuzbein
L89.37	Dekubitus 4. Grades : Ferse
L97	Ulcus cruris, anderenorts nicht klassifiziert
M00.97	Eitrige Arthritis, nicht näher bezeichnet : Knöchel und Fuß [Fußwurzel, Mittelfuß, Zehen, Sprunggelenk, sonstige Gelenke des Fußes]
M14.6	Neuropathische Arthropathie
M21.68	Sonstige erworbene Deformitäten des Knöchels und des Fußes
M24.56	Gelenkkontraktur : Unterschenkel [Fibula, Tibia, Kniegelenk]
M60.05	Infektiöse Myositis : Beckenregion und Oberschenkel [Becken, Femur, Gesäß, Hüfte, Hüftgelenk, Iliosakralgelenk]
M62.25	Ischämischer Muskelinfarkt (nichttraumatisch) : Beckenregion und Oberschenkel [Becken, Femur, Gesäß, Hüfte, Hüftgelenk, Iliosakralgelenk]
M62.26	Ischämischer Muskelinfarkt (nichttraumatisch) : Unterschenkel [Fibula, Tibia, Kniegelenk]
M62.27	Ischämischer Muskelinfarkt (nichttraumatisch) : Knöchel und Fuß [Fußwurzel, Mittelfuß, Zehen, Sprunggelenk, sonstige Gelenke des Fußes]
M72.65	Nekrotisierende Fasziitis : Beckenregion und Oberschenkel [Becken, Femur, Gesäß, Hüfte, Hüftgelenk, Iliosakralgelenk]
M72.66	Nekrotisierende Fasziitis : Unterschenkel [Fibula, Tibia, Kniegelenk]
M72.67	Nekrotisierende Fasziitis : Knöchel und Fuß [Fußwurzel, Mittelfuß, Zehen, Sprunggelenk, sonstige Gelenke des Fußes]
M79.99	Krankheit des Weichteilgewebes, nicht näher bezeichnet : Nicht näher bezeichnete Lokalisation
M84.07	Frakturheilung in Fehlstellung : Knöchel und Fuß [Fußwurzel, Mittelfuß, Zehen, Sprunggelenk, sonstige Gelenke des Fußes]

M86.16	Sonstige akute Osteomyelitis : Unterschenkel [Fibula, Tibia, Kniegelenk]
M86.17	Sonstige akute Osteomyelitis : Knöchel und Fuß [Fußwurzel, Mittelfuß, Zehen, Sprunggelenk, sonstige Gelenke des Fußes]
M86.46	Chronische Osteomyelitis mit Fistel : Unterschenkel [Fibula, Tibia, Kniegelenk]
M86.47	Chronische Osteomyelitis mit Fistel : Knöchel und Fuß [Fußwurzel, Mittelfuß, Zehen, Sprunggelenk, sonstige Gelenke des Fußes]
M86.67	Sonstige chronische Osteomyelitis : Knöchel und Fuß [Fußwurzel, Mittelfuß, Zehen, Sprunggelenk, sonstige Gelenke des Fußes]
M86.87	Sonstige Osteomyelitis : Knöchel und Fuß [Fußwurzel, Mittelfuß, Zehen, Sprunggelenk, sonstige Gelenke des Fußes]
M86.96	Osteomyelitis, nicht näher bezeichnet : Unterschenkel [Fibula, Tibia, Kniegelenk]
M86.97	Osteomyelitis, nicht näher bezeichnet : Knöchel und Fuß [Fußwurzel, Mittelfuß, Zehen, Sprunggelenk, sonstige Gelenke des Fußes]
M87.97	Knochennekrose, nicht näher bezeichnet : Knöchel und Fuß [Fußwurzel, Mittelfuß, Zehen, Sprunggelenk, sonstige Gelenke des Fußes]
N08.3	Glomeruläre Krankheiten bei Diabetes mellitus
N17.0	Akutes Nierenversagen mit Tubulusnekrose
N18.5	Chronische Nierenkrankheit, Stadium 5
N19	Nicht näher bezeichnete Niereninsuffizienz
N39.0	Harnwegsinfektion, Lokalisation nicht näher bezeichnet
Q85.0	Neurofibromatose (nicht bösartig)
R02	Gangrän, anderenorts nicht klassifiziert
R57.0	Kardiogener Schock
R57.2	Septischer Schock
R57.8	Sonstige Formen des Schocks - Inkl.: Endotoxinschock
R65.0	Systemisches inflammatorisches Response-Syndrom [SIRS] infektiöser Genese ohne Organkomplikationen
R65.1	Systemisches inflammatorisches Response-Syndrom [SIRS] infektiöser Genese mit Organkomplikationen
S06.4	Epidurale Blutung
S32.4	Fraktur des Acetabulums
S35.5	Verletzung von Blutgefäßen der Iliakalregion
S40.0	Prellung der Schulter und des Oberarmes
S51.86	Weichteilschaden III. Grades bei geschlossener Fraktur oder Luxation des Unterarmes
S52.4	Fraktur des Ulna- und Radiusschaftes, kombiniert
S70.81	Oberflächliche Verletzung der Hüfte und des Oberschenkels - Schürfwunde
S71.0	Offene Wunde der Hüfte
S71.1	Offene Wunde des Oberschenkels
S71.88	Weichteilschaden II. Grades bei offener Fraktur oder Luxation der Hüfte und des Oberschenkels
S72.10	Pertrochantäre Fraktur - Trochantär, nicht näher bezeichnet
S72.3	Fraktur des Femurschaftes
S80.1	Prellung sonstiger und nicht näher bezeichneter Teile des Unterschenkels
S80.88	Oberflächliche Verletzung des Unterschenkels - Sonstige
S81.7	Multiple offene Wunden des Unterschenkels
S81.80	Nicht näher bezeichnete offene Wunde sonstiger Teile des Unterschenkels
S81.85	Weichteilschaden II. Grades bei geschlossener Fraktur oder Luxation des Unterschenkels

S81.88	Weichteilschaden II. Grades bei offener Fraktur oder Luxation des Unterschenkels
S81.89	Weichteilschaden III. Grades bei offener Fraktur oder Luxation des Unterschenkels
S81.9	Offene Wunde des Unterschenkels, Teil nicht näher bezeichnet
S82.18	Fraktur des Unterschenkels, einschließlich des oberen Sprunggelenkes - Sonstige
S82.21	Fraktur des Tibiaschaftes - Mit Fraktur der Fibula (jeder Teil)
S82.28	Fraktur des Tibiaschaftes - Sonstige
S82.31	Distale Fraktur der Tibia - Mit Fraktur der Fibula (jeder Teil)
S82.82	Frakturen sonstiger Teile der Unterschenkels - Trimalleolarfraktur
S82.9	Fraktur des Unterschenkels, Teil nicht näher bezeichnet
S83.10	Luxation, Verstauchung und Zerrung des Kniegelenkes und von Bändern des Kniegelenkes - Nicht näher bezeichnet
S85.0	Verletzung der A. poplitea
S86.7	Verletzung mehrerer Muskeln und Sehnen in Höhe des Unterschenkels
S88.9	Traumatische Amputation am Unterschenkel, Höhe nicht näher bezeichnet
S91.1	Offene Wunde einer oder mehrerer Zehen ohne Schädigung des Nagels
S91.3	Offene Wunde sonstiger Teile des Fußes
S91.86	Offene Wunde sonstiger Teile der Knöchelregion und des Fußes - Weichteilschaden III. Grades bei geschlossener Fraktur oder Luxation des Fußes
S91.89	Offene Wunde sonstiger Teile der Knöchelregion und des Fußes - Weichteilschaden III. Grades bei offener Fraktur oder Luxation des Fußes
S92.20	Fraktur - Ein oder mehrere sonstige Fußwurzelknochen, nicht näher bezeichnet
S92.3	Fraktur der Mittelfußknochen
S92.5	Fraktur einer sonstigen Zehe
S93.33	Luxation - Tarsometatarsal (-Gelenk)
S96.1	Verletzung von Muskeln und Sehnen der langen Streckmuskeln der Zehen in Höhe des Knöchels und des Fußes
S98.0	Traumatische Amputation des Fußes in Höhe des oberen Sprunggelenkes
S98.1	Traumatische Amputation einer einzelnen Zehe
S98.3	Traumatische Amputation sonstiger Teile des Fußes
T25.3	Verbrennung 3. Grades der Knöchelregion und des Fußes
T31.00	Verbrennungen von weniger als 10% der Körperoberfläche : Weniger als 10% oder nicht näher bezeichneter Anteil von Verbrennungen 3. Grades
T79.6	Traumatische Muskelischämie
T79.62	Traumatische Muskelischämie des Unterschenkels
T81.3	Komplikationen bei Eingriffen - Aufreißen einer Operationswunde, anderenorts nicht klassifiziert
T81.4	Komplikationen bei Eingriffen - Infektion nach einem Eingriff, anderenorts nicht klassifiziert
T81.7	Komplikationen bei Eingriffen - Gefäßkomplikationen nach einem Eingriff, anderenorts nicht klassifiziert
T82.7	Infektion und entzündliche Reaktion durch sonstige Geräte, Implantate oder Transplantate im Herzen und in den Gefäßen
T82.8	Sonstige näher bezeichnete Komplikationen durch Prothesen, Implantate oder Transplantate im Herzen und in den Gefäßen
T84.5	Infektion und entzündliche Reaktion durch eine Gelenkendoprothese
T84.6	Infektion und entzündliche Reaktion durch eine interne Osteosynthesevorrichtung [jede Lokalisation]
T87.3	Neurom des Amputationsstumpfes
T87.4	Infektion des Amputationsstumpfes

T87.5	Nekrose des Amputationsstumpfes
T87.6	Sonstige und nicht näher bezeichnete Komplikationen am Amputationsstumpf
T89.00	Komplikationen einer offenen Wunde - Nicht näher bezeichnet
T95.3	Folgen einer Verbrennung, Verätzung oder Erfrierung der unteren Extremität
U80.00	Staphylococcus aureus mit Resistenz gegen Oxacillin oder Methicillin [MRSA]
Z47.0	Entfernung einer Metallplatte oder einer anderen inneren Fixationsvorrichtung
Z89.6	Verlust der unteren Extremität oberhalb des Knies, einseitig
Z95.0	Vorhandensein eines kardialen elektronischen Geräts
Z95.5	Vorhandensein eines Implantates oder Transplantates nach koronarer Gefäßplastik
Z95.88	Vorhandensein von sonstigen kardialen oder vaskulären Implantaten oder Transplantaten
Z96.6	Vorhandensein von orthopädischen Gelenkimplantaten
Z96.65	Vorhandensein einer Kniegelenkprothese

Supplementary Data S1: All ICD-Codes

ICD-Codes	Diagnosis
I70.2-	Atherosclerosis of arteries of extremities
J15.-	Pneumonia
J44.-	Other chronic obstructive pulmonary disease
J90.-	Pleural effusion
I21.-	Acute myocardial infarction
I49.-	Other cardiac arrhythmias
I10.-	Hypertension
I25.-	Chronic Ischaemic Heart Disease
T82.-	Complications of cardiac and vascular prosthetic devices, implants and grafts
F06.-	Other mental disorders due to brain damage and dysfunction and to physical disease
I70.8	Atherosclerosis
D68.-	Other coagulation defects
K76.-	Other diseases of liver
I79.-	Disorders of arteries, arterioles and capillaries in diseases classified elsewhere
Z99.-	Dependence on renal dialysis
R02.-	Gangrene
I87.21	Venous insufficiency with ulceration
M62.2	Ischaemic infarction of muscle
L89.-	Decubitus ulcer and pressure area
I82.- / I74.-	Other venous / arterial embolism and thrombosis
F10.-	Mental and behavioural disorders due to use of alcohol
T65.2	Toxic effect: Tobacco and Nicotine
E10.-/E11.-/E14.-	Diabetes Mellitus
G63.-	Polyneuropathy in diseases classified elsewhere
A41.- / R65.-	Sepsis / SIRS
L03.-	Cellulitis
M72.6-	Necrotizing fasciitis
M86.-	Osteomyelitis
A49.-	Bacterial infection of unspecified site
T81.-	Complications of procedures
E78.5	Hyperlipidaemia
E66.0-	Adipositas

Supplementary Data S2: Included ICD-Codes

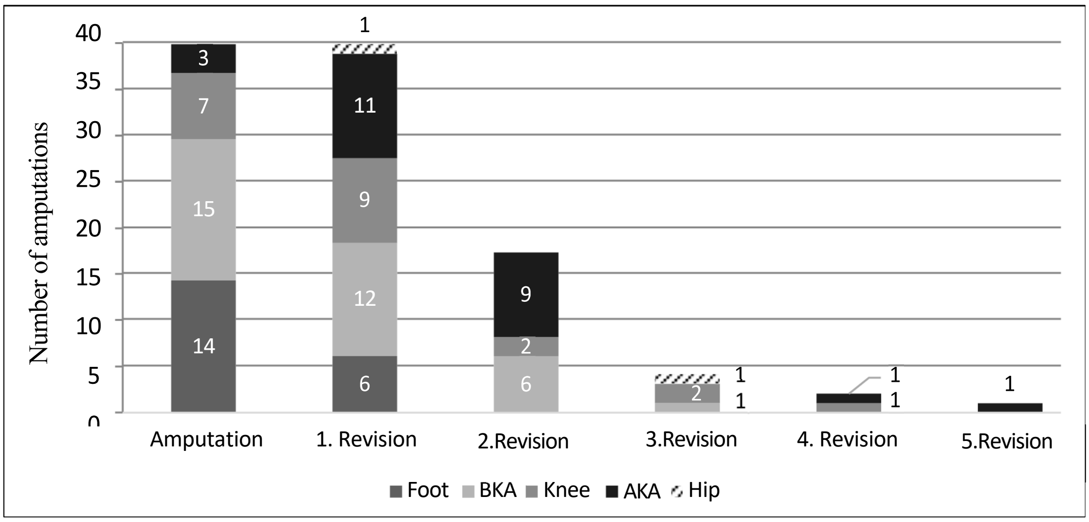

Supplementary Data S3. Amputation levels from initial non traumatic amputation to fifth revision.
